# Treatment of Spermatorrhœa by a Double Truss

**Published:** 1875-03

**Authors:** 


### TREATMENT OF SPERMATORRHŒA BY A DOUBLE TRUSS.

In the Spring of 1867, I was consulted by a gentleman, of the age of 32, under the following circumstances: He had been married three months. He was in good health, well-formed and muscular. The sexual desire was very feeble; there was no erection, and he had not been able to have connection with his wife. There were no nocturnal emissions. Before marriage he had been strictly continent. The genitals were normal, and well-developed. He was wearing a double truss, the pads of which were well applied to the external ring, and pressed on the cords. In boyhood he had inguinal hernia of the right side, for which the double truss was then ordered, and he had worn it carefully ever since. I could find no trace of the rupture, nor had he seen anything of it for two or three years. I suspected that the pressure on the cord checked the passage of the fluid along the duct to the seminal vesicle, and had gradually caused more or less complete suspension of the secretion in the testicle, or its absorption there. I advised him to remove the truss, to watch carefully for the reappearance of the rupture, and to wait patiently for the manifestation of sexual desire and power. He was not disappointed, for these were slowly but steadily developed, and in about six weeks he was able to have intercourse. As time went on, the improvement became complete, and he is now the father of three children.

This case suggested to me the employment of the double truss in the treatment of spermatorrhœa; and I have used it in several cases with signal advantage. The mention of one of these cases will suffice. It was that of a young man, aged 27, of a spare habit of body and highly nervous temperament. From the age of twenty to twenty four he had indulged, very moderately, in sexual intercourse, and had not had any sexual disorder. About this time he began to suffer from irritable dyspepsia and constipation, and ever since had been severely troubled with these ailments; and, in connection with them, nocturnal emisions had been manifested. These now occurred every second or third night, often more frequently, which exhausted him greatly, and rendered his life miserable.

Along with other means for the dyspepsia constipations and general health, I directed the use of the double truss, so as to exercise a gentle pressure on the cords both night and day.

Improvement was quickly manifested; the emissions recurred with less and less frequency, and, after a few months, showed themselves only at intervals of one or two weeks; the general health likewise improved steadily. He wore the truss for about a year; and, though still delicate, has continued since in fair general health and freedom from spermatorrhœa.

I believe that in this case the spermatorrhœa was the result of the dyspepsia and constipation, conjoined with a highly nervous temperament; the undue frequency of seminal emissions, in their turn, intensifying the nervous exhaustion, and aggravating the functional disorder of stomach and bowels—a vicious circle, which embittered the life of the unhappy patient.

This has been the usual feature of the cases which have come under my care, and I have less experience of the ailment occurring in healthy young men in connection with the disease of the urethra; but in these cases I have no doubt that the double truss would help very materially in the cure of the disorder. The pressure of the truss should be quite gentle; and, thus applied, there is abundant evidence to show that permanent impairment of sexual vigor need not be apprehended.—W. F., *in the British Medical Journal.*

---

*Silica in Cancer.*—In the November number of the Edinburg *Med. Jour.* Mr. Fawcett Battye narrates his experience with an entirely new remedy in cancer. This is silica, powdered very fine, and administered internally twice or thrice a day, in one-grain doses, combined with a third of a grain of morphia. He found it to diminish the pain in a very marked degree, and by the tenth day to disperse it altogether. He does not precisely claim, however, that the patients recovered. They were relieved and benefited, and when they took it continuously, the disease was retarded. No satisfactory explanation of its action is advanced.—*Med. and Surg. Rep.*

